# Tyrosine Hydroxylase Is Short-Term Regulated by the Ubiquitin-Proteasome System in PC12 Cells and Hypothalamic and Brainstem Neurons from Spontaneously Hypertensive Rats: Possible Implications in Hypertension

**DOI:** 10.1371/journal.pone.0116597

**Published:** 2015-02-24

**Authors:** Nadia A. Longo Carbajosa, Gerardo Corradi, María A. Lopez Verrilli, María J. Guil, Marcelo S. Vatta, Mariela M. Gironacci

**Affiliations:** 1 Departamento de Química Biológica, IQUIFIB-CONICET, Facultad de Farmacia y Bioquímica, Universidad de Buenos Aires, Buenos Aires, Argentina; 2 Cátedra de Fisiología, IQUIMEFA-CONICET, Facultad de Farmacia y Bioquímica, Universidad de Buenos Aires, Buenos Aires, Argentina; University of Buenos Aires, Faculty of Medicine. Cardiovascular Pathophysiology Institute., ARGENTINA

## Abstract

Aberrations in the ubiquitin-proteasome system (UPS) are implicated in the pathogenesis of various diseases. Tyrosine hydroxylase (TH), the rate-limiting enzyme in catecholamines biosynthesis, is involved in hypertension development. In this study we investigated whether UPS regulated TH turnover in PC12 cells and hypothalamic and brainstem neurons from spontaneously hypertensive rats (SHR) and whether this system was impaired in hypertension. PC12 cells were exposed to proteasome or lysosome inhibitors and TH protein level evaluated by Western blot. Lactacystin, a proteasome inhibitor, induced an increase of 86±15% in TH levels after 30 min of incubation, then it started to decrease up to 6 h to reach control levels and finally it rose up to 35.2±8.5% after 24 h. Bafilomycin, a lysosome inhibitor, did not alter TH protein levels during short times, but it increased TH by 92±22% above basal after 6 h treatment. Before degradation proteasome substrates are labeled by conjugation with ubiquitin. Efficacy of proteasome inhibition on TH turnover was evidenced by accumulation of ubiquitinylated TH after 30 min. Further, the inhibition of proteasome increased the quantity of TH phosphorylated at Ser40, which is essential for TH activity, by 2.7±0.3 fold above basal. TH protein level was upregulated in neurons from hypothalami and brainstem of SHR when the proteasome was inhibited during 30 min, supporting that neuronal TH is also short-term regulated by the proteasome. Since the increased TH levels reported in hypertension may result from proteasome dysfunction, we evaluate proteasme activity. Proteasome activity was significantly reduced by 67±4% in hypothalamic and brainstem neurons from SHR while its protein levels did not change. Present findings show that TH is regulated by the UPS. The impairment in proteasome activity observed in SHR neurons may be one of the causes of the increased TH protein levels reported in hypertension.

## Introduction

Cellular protein degradation is a highly complex, temporally controlled, and tightly regulated process that plays a critical role in a variety of basic pathways not only during cell life and death but also in health and disease. The ubiquitin-proteasome system (UPS) is the major pathway for intracellular protein degradation in eukaryotic cells [1,2]. Degradation of proteins by the UPS occurs in two successive steps: 1) conjugation of multiple ubiquitin (Ub) moieties to the substrate and 2) degradation of the tagged protein by the downstream 26S proteasome complex. This complex is composed of a 20S core particle which embodies the catalytic activity and two 19S regulatory particles [1,2]. Attachment of Ub is the dominant mechanism to tag proteins for degradation by the 26S proteasome and involves the activity of three types of enzymes: E1 ubiquitin–activating enzyme, E2 ubiquitin–carrier enzyme and E3 ubiquitin–protein ligase. The sequential action of these enzymes leads to conjugation of Ub to proteins. Different E3 ligase target specific substrates for degradation and its activity could be under local control depending on the presence of regulatory cofactors [1,3–5].

Alterations in the UPS are implicated in the pathogenesis of cancer, neurodegenerative and immune diseases [1–5] and further, the UPS has been recognized as a key regulatory pathway in cardiovascular diseases [6–8]. Recent evidence also shows that the UPS modulates the activity of endothelial nitric oxide synthase, the major enzyme involved in vascular homeostasis which by interacting with other vasoactive mediators and influencing the oxidative stress response in the vasculature contributes to the regulation of endothelial (dys)function [7–9].

L-tyrosine hydroxylase (EC 1.14.16.2) (TH) is the first enzyme and rate-limiting step in catecholamine biosynthesis that catalyzes the conversion of L-tyrosine to L-dihydroxyphenylalanine [10]. Increased catecholaminergic neurotransmission has been reported in spontaneously hypertensive rats (SHR), characterized by increased TH activity as well as gene and protein expression [11–13], suggesting that TH plays a key role in genesis, development and/or maintenance of hypertension. In fact, Rao et al. [14] have reported that common variation in the TH proximal promoter contributes to inheritable alteration in multiple autonomic traits, biochemical and physiological, and the ultimate disease trait of hypertension.

TH activity is regulated by two mechanisms: short-term direct regulation of enzyme activity (allosteric regulation, catecholamin feedback inhibition and phosphorylation) and medium- to long-term regulation of gene transcription [10]. In addition, enzyme activity is also regulated through its turnover. The half-life of rat TH has been reported to be 17 h [15], 30 h [16] and 29 h [17] in PC12 cells, in a subclone of PC12 cells and in chromaffin cells, respectively. In addition, Nakashima et al. [18] reported that no TH degradation by the proteasome occurs in PC12 cells at times around 8 h, but times smaller than 4 h were not evaluated. Our aim was to investigate whether TH is short-term modulated by the UPS. Given that aberrations in the UPS are implicated in the pathogenesis of many diseases, and that TH protein levels and activity are augmented in SHR, which may be a consequence of impairment in the UPS activity, we investigated proteasome activity and protein levels centrally in SHR.

## Materials and Methods

### Reagents

Fetal bovine serum, penicilin-streptomycin, goat anti-mouse antibody coupled to Alexa 594, goat anti-rabbit antibody coupled to Alexa 488 and Dulbecco’s modified Eagle’s medium (DMEM) were purchased from Invitrogen (Carlsbad, CA, USA). Rabbit anti-Ub antibody was from Santa Cruz Biotechnology Laboratories (Santa Cruz, CA, USA). Bovine seroalbumin (BSA), paraformaldehyde (PFA 4%), phosphate buffered saline (PBS) and the protease inhibitors cocktail were from Sigma Chemical Co. (St. Louis, MO, USA). Polyclonal anti-TH rabbit, monoclonal anti-TH mouse, anti-TH phosphoSer-19 rabbit and anti-TH phosphoSer-40 rabbit antibodies were from Chemicon (San Francisco, CA, USA). Phosphatase inhibitor, ubiquitinated protein enrichment kit, proteasome substrate III Suc-Leu-Leu-Val-Tyr-aminomethyl coumarin (Suc-LLVY-AMC) and monoclonal anti-20S proteasome alfa1,2,3,5,6&7-subunits mouse antibody were from Calbiochem (EMD Chemicals, Gibbstown, USA).

### Animals

Three-months old male Wistar-Kyoto (WKY) and SHR rats were used in the present work. The animals were housed in cages, with a 12-hour light/dark cycle, controlled temperature and humidity. All animals were given water and food ad libitum (commercial rodents Purina chow, Cooperacion SRL, Argentina).

### Mean arterial blood pressure measurement

A guide cannula was placed in the rat brain left lateral ventricle. Briefly, animals under anaesthesia (50 mg/kg ketamine plus 10 mg/kg xylazine) were mounted in a stereotaxic apparatus (Kopf model 900, David Kopf Instruments, USA) and a small hole was drilled through the skull. An intracranial cannula was placed into the left lateral ventricle by using appropriate stereotaxic coordinates [19]. The cannula was secured by two screws inserted into the surface of the bone using cyanoacrylate. Rats were placed in individual cages and allowed to recover from surgery. Seven days after surgery, WKY and SHR rats were anesthetized with urethane (1.2 g/kg of body weight) and the femoral artery cannulated. A catheter was connected to a pressure transducer and signals recorded in a AD Instruments apparatus (Power Lab 8/30). Following 20 min stabilization period, artificial cerebrospinal fluid (aCSF) or MG-132 (20 μM, 1 μl/min) was injected in the lateral ventricle and systolic and diastolic pressures as well as cardiac rate were recorded for 60 min. MG-132 is an agent substrate analogues and potent transition-state inhibitor primarily of the chymotrypsin-like activity of the proteasome, reversible and with high cell penetration [20]. The dose of MG-132 was chosen according to previous studies [21].

### Cell culture

PC12 cells, a rat adrenal pheochromocytoma cell line which expresses catecholaminergic neuronal properties [22], were grown in DMEM high glucose supplemented with 5% heat inactivated fetal bovine serum, 10% horse serum and penicilin-streptomycin at 37 C in a humidified atmosphere at 95% air and 5% CO_2_. Cells were serum-starved 24 h prior to treatment. PC12 cells were a gift from Dr. Sandra Verstraeten who acquired them from ATTC (USA).

Neuronal cultures were obtained from hypothalamus-brainstem areas of 1-day-old WKY and SHR rats and cells were used after 8–10 days in culture as previously reported [12].

Cell viability was evaluated in cells exposed to different treatments as describe by Uludag and Sefton [23].

The proteasome inhibitor lactacystin was used at a concentration of 1 or 5 μmol/L as previously reported [12].

### TH protein measurement

TH protein level was measured by Western blot [12]. Membranes were subsequently probed with either mouse anti-TH (1:500) to measure TH protein level or with rabbit anti-TH phosphoSer-19 (1:300) or anti-TH phosphoSer-40 (1:300) to meassure TH phosphorylation, followed by incubation with goat anti-mouse or goat anti-rabbit IgGs coupled to horseradish peroxidase (Amersham Biosciences, Piscataway, NY, USA). Immunoreactive bands were visualized by chemiluminescence detection (ECL Plus reagent, Amersham Biosciences, Piscataway, NY, USA) and quantified by densitometry. Protein loading was evaluated by reblotting membranes with anti-GAPDH antibody.

### TH ubiquitination

Cells were incubated in the absence or presence of the proteasome inhibitor and Ub-TH was isolated with an ubiquitylated protein enrichment kit which binds ubiquitylated cellular proteins (Calbiochem). Proteins were resolved in a 4–12% SDS-PAGE and Ub-TH protein level was evaluated by Western-blot as described above using a mouse anti-TH antibody (dilution 1/500). Membranes were reprobed with a rabbit anti-Ub antibody (dilution 1/500) to further confirm the efectiveness of the ubiquitylated protein enrichment kit.

### Immunocytochemistry

Cells were fixed in 4% PFA and immunostained as previously described [24]. Cells were incubated with either rabbit anti-TH antibody (1:500) plus mouse anti-proteasome antibody (1:250) or mouse anti-TH antibody (1:1000) plus rabbit anti-Ub antibody (1:150) or either rabbit anti-TH phosphoSer-19 (1:300) or anti-TH phosphoSer-40 anti-TH antibody (1:500) plus mouse anti-proteasome antibody (1:250) in PBS-T 90 min at room temperature. Cells were incubated with anti-rabbit antibody coupled to Alexa 488 (1:500) and anti-mouse IgG AlexaFluor 594 (1:600) for 1 h at room temperature. Nonspecific staining was measured in cells treated with blocking solution in the absence of the primary antibody. Samples were mounted and imaged using an Olympus Fluoview FV1000 spectral laser scanning confocal microscope with a 60x oil immersion lens using dual excitation (473 nm for Alexa 488 and 559 nm for Alexa 594). Due to the spectral properties of the scan head, fluorescence emission was collected between 510 and 550 nm for Alexa 488 and 600–660 nm for Alexa 594. Images were obtained using sequential scanning for each channel to eliminate the cross-talk of chromophores.

Quantitative colocalization was estimated by Pearson´s correlation coefficient and overlap coefficient according to Manders [25], which were calculated using Image-Pro Plus software (MediaCybernetics Inc.).

### 26S Proteasome activity

Proteasome activity was evaluated in the hypothalamus and brainstem as well as in primary neuronal cultures from these brain areas of WKY or SHR rats. The study was performed in these brain areas given their relevance in the control of cardiovascular function, and further we have previously reported that angiotensin-(1–7) regulates TH expression through the UPS system in neuronal cultures from rat hypothalamus and brainstem [12]. Rats were euthanized by decapitation and the hypothalamus and brainstem removed. Tissues or neuronal cells were homogenized in ice cold buffer (pH 7.4) containing 25 mmol/L Hepes, 1 mmol/L EDTA, 0.32 mol/L sucrose and centrifuged at 14000 xg at 4°C for 15 min. The supernatant was used to assay 26S proteasome activity by measuring ATP-dependent degradation of the proteasome fluorescence substrate III Suc-LLVY-AMC as previously described [26]. Briefly, lysates were incubated at 37°C in incubation buffer (50 mmol/L Tris-HCl, pH 7.5, 40 mmol/L KCl, 5 mmol/L MgCl2, 1.1 mM DTT, 0.5 mg/mL BSA and 0.5 mmol/L ATP), and 0.1 mmol/L Suc-Leu-Leu-Val-Tyr AMC. AMC hydrolysis was quantified in a spectrofluorometer (Aminco Bowman Series 2) at 380 nm excitation and 460 nm emission wavelengths. Enzymatic activity was normalized to protein concentration and expressed as % of change with respect to the enzymatic activity in WKY rats.

### Proteasome protein level measurement

Proteasome protein level was measured by Western blot in the hypothalamus and brainstem as well as in primary neuronal cultures from the hypothalamus and brainstem of WKY or SHR rats as described above using a monoclonal anti-20S proteasome alfa1,2,3,5,6&7-subunits mouse antibody (Calbiochem) (1/1000).

### Ethics Statement

This study was carried out in strict accordance with the recommendations in the Guide for the Care and Use of Laboratory Animals (NIH Publication No. 85-23, 1985, revised 1996). The protocol was approved by the Institutional Animals Care and Use Committee of the School of Pharmacy and Biochemistry, University of Buenos Aires, Argentina (permit number 031013-5).

### Statistical analysis

The data are presented as mean ± SEM. Statistical significance was assessed by Student-t-test or one-way analysis of variance (ANOVA) followed by Bonferroni post tests (GraphPad Prism 4, GraphPad Software, Inc.). P values <0.05 were considered statistically significant.

## Results

### TH is short-term regulated by the UPS

**Fig 1:**
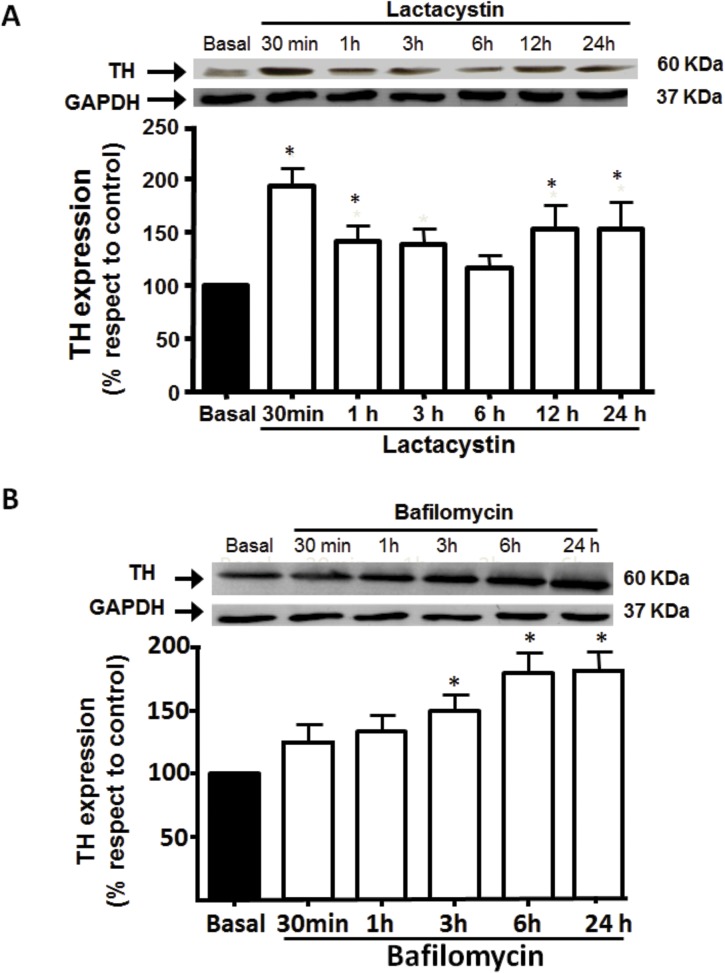
Effect of proteasome and lysosome inhibition on TH protein level. PC12 cells were treated with 1 μmol/L lactacystin (A), a specific proteasome inhibitor, or 5 nmol/L bafilomycin (B), a H^+^-ATPase inhibitor, at different times and TH protein level assessed by Western-blot as described under Methods. The blots were stripped and reprobed for GAPDH to assess protein loading. Signals from TH protein level were normalized to GAPDH levels. Each bar represents the mean ± SEM of 3 determinations from four separate cell culture preparations. * P < 0.05 as compared to basal.

Fig. 1A shows the effect of lactacystin, a specific proteasome inhibitor, on TH levels in PC12 cells. Time dependent experiments with 1 μmol/L lactacystin showed that TH increased by 86±15% at 30 min, then decreased to reach basal levels at 6 h but then augmented up to 45.2±8.5% following 24 h (Fig. 1A). These findings suggest that proteasome regulates TH protein level, at short and long time periods.

As the decrease in TH at 6 h may involve another degradating pathway such as lysosomes, cell were exposed to bafilomycin, an inhibitor of H^+^-ATPase, the enzyme that maintains the acidic pH within lysosomes. Bafilomycin (5 nmol/L) did not alter TH protein content at short times, but it significantly increased the enzyme by 92±22% after 6 h treatment (Fig. 1B).

None of the treatments affected cell viability before 12 h but after 24 h incubation with the proteasome inhibitor cell viability declined 35±5% (data not shown).

**Fig 2:**
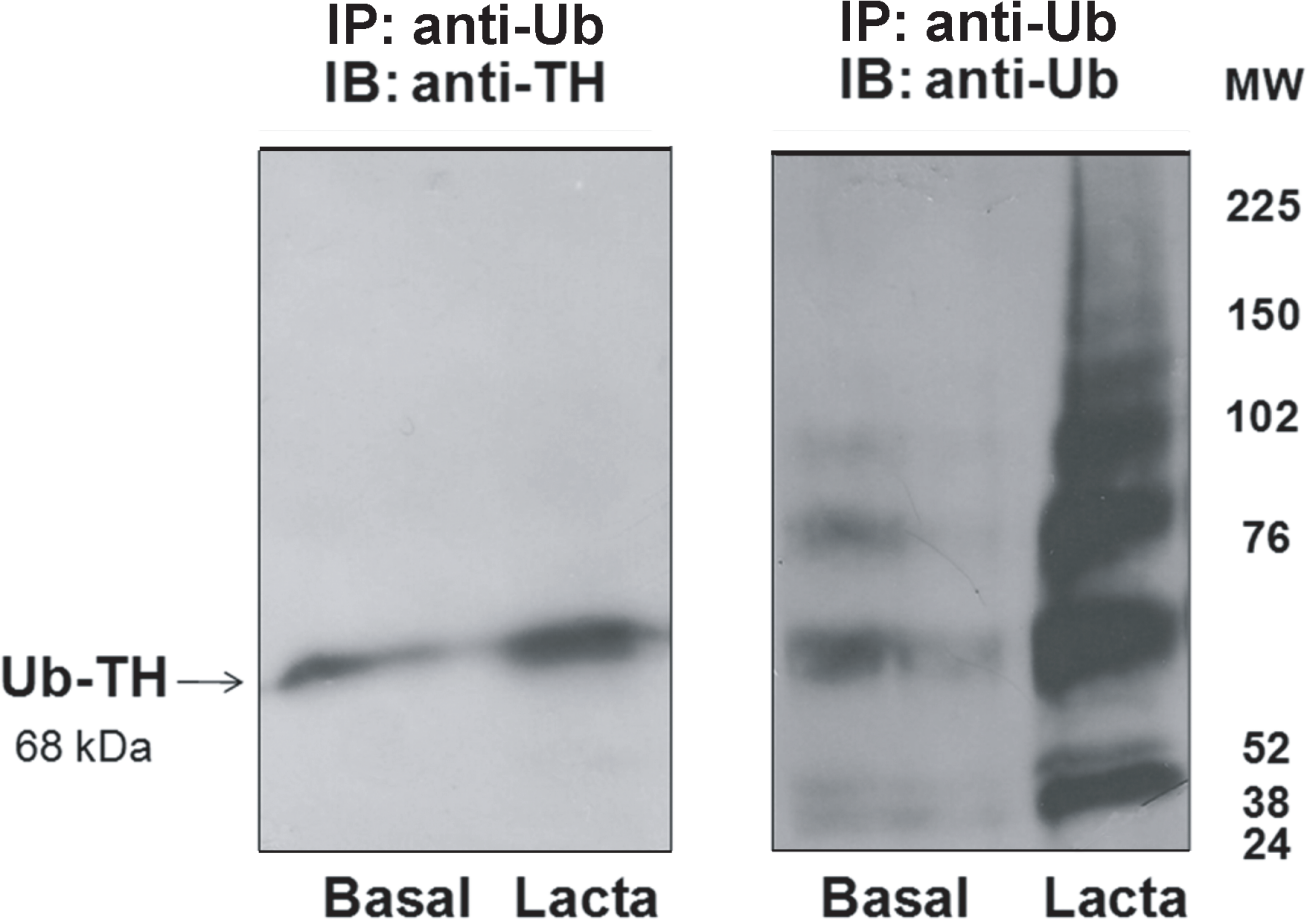
TH ubiquitination. PC12 cells were incubated for 30 min in the absence (basal) or presence of lactacystin (Lacta) (1 μmol/L). Ub-TH was immunoprecipitated with an ubiquitinated protein enrichment kit (IP: anti-Ub). Proteins were resolved in a 4–12% SDS-PAGE and Ub-TH protein level assessed by Western-blot as described under Methods. The same membrane was stripped and reprobed with an antibody against ubiquitin (Ub) to further corroborate that Ub-proteins were isolated with the kit. Three separate experiments were performed. A representative Western blot immunoblotted with antibodies directed against TH or Ub is shown.

**Fig 3:**
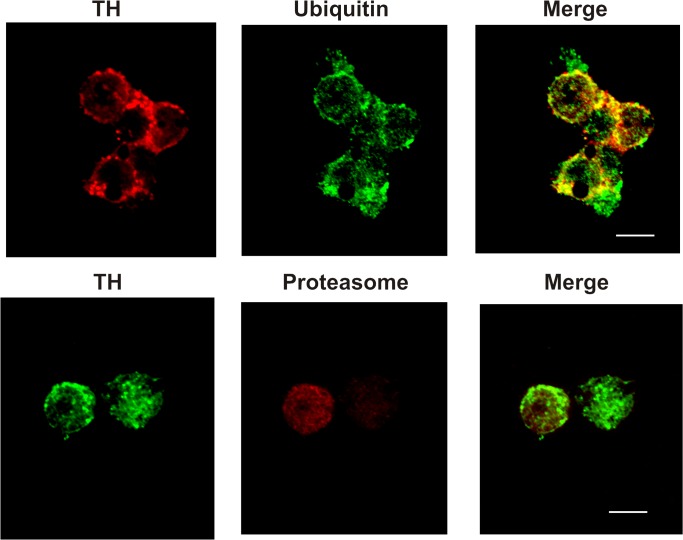
Colocalization of TH with ubiquitin or proteasome in PC12 cells. Cells were incubated with either mouse anti-TH plus rabbit anti-ubiquitin antibodies (upper panel) or rabbit anti-TH plus mouse anti-proteasome antibodies (lower panel) followed by incubation with a anti-rabbit antibody coupled to Alexa 488 and anti-mouse coupled to AlexaFluor 594. See text for details. Scale bar = 5 μm. Three separate cell culture preparations were performed and 10 different images were taken and analyzed for each experiment. One representative image is presented.

Proteasome can degrade poly- [3] or mono- [27,28] or even unubiquitylated proteins [29]. As proteasome substrates are generally tagged with ubiquitin before degradation [3], we addressed whether TH was ubiquitylated before it was recognized by the proteosome. PC12 cells were incubated in the presence or absence of lactacystin during 30 min and Ub-TH content was determined. We focused our study at a short time (30 min) because TH turnover at longer times (more than 12 h) have been investigated [15–17]. As it is shown in Fig. 2, TH was monoubiquitylated and the efficacy of proteasome inhibition on TH turnover in PC12 cells was evidenced by ubiquitylated TH accumulation after exposure to the proteasome inhibitor for 30 min, supporting that TH is ubiquitylated before being degraded by the proteasome. To further confirm this result, the colocalization of TH with either Ub or the proteasome 26S was evaluated. Fig. 3 shows that TH colocalized with both Ub and proteasome.

**Fig 4:**
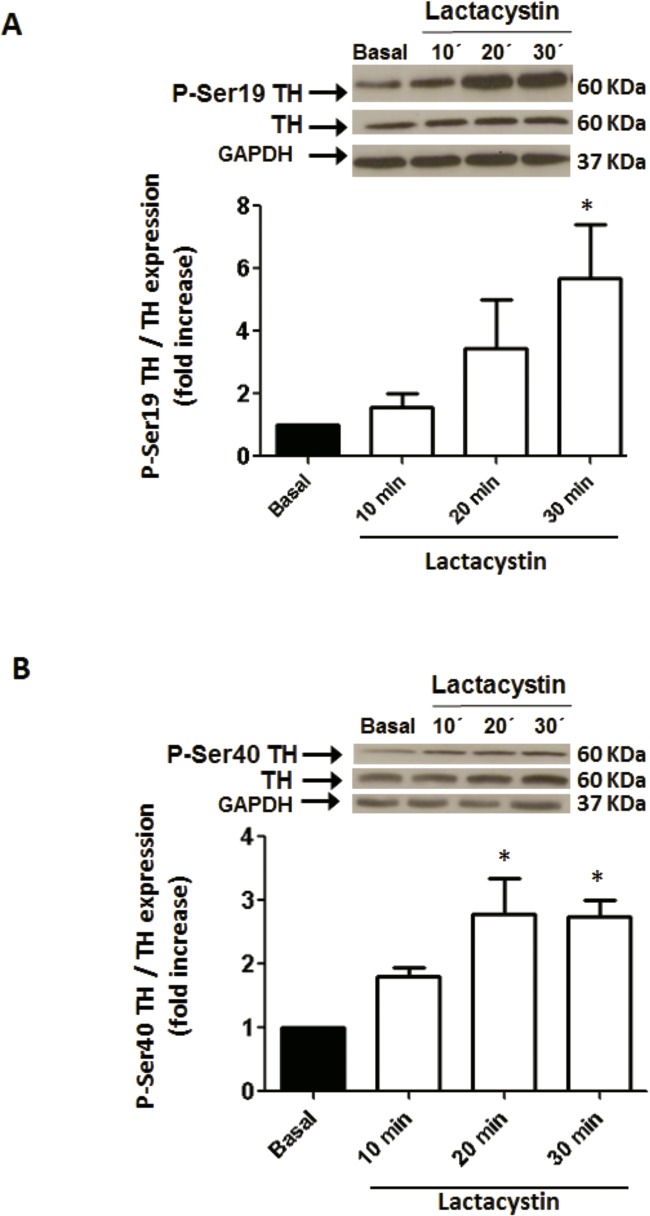
Effect of proteasome inhibition on TH phosphorylation at Ser19 (A) and Ser40 (B). PC12 cells were exposed to 1 μmol/L lactacystin at different times and TH-phospho Ser19 or TH-phospho Ser40 were measured by Western-blot as described in Methods and normalized to TH protein level in the same sample. Protein loading was evaluated by reblotting the membrane with anti-GAPDH antibody and no differences were observed in any of the conditions assayed. Each bar represents the mean ± SEM of 3 determinations from four separate cell culture preparations. * P < 0.05 as compared to basal.

**Fig 5:**
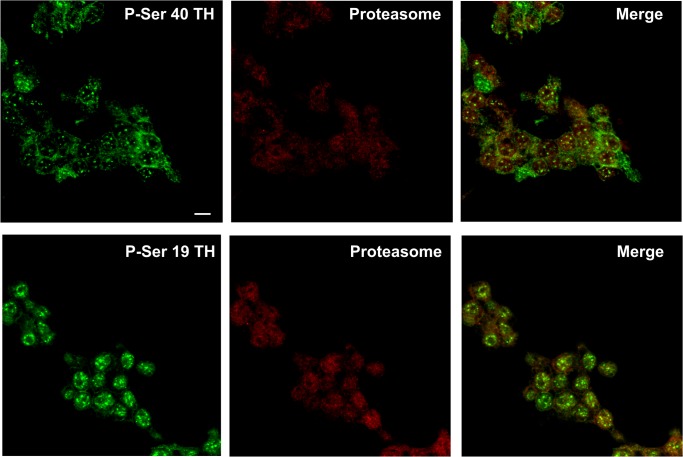
Colocalization of phospho-Ser40-TH (P-Ser 40 TH) or phospho-Ser19-TH (P-Ser 19 TH) with proteasome in PC12 cells. Cells were incubated with either rabbit phospho-Ser40-TH plus mouse proteasome antibodies (upper panel) or rabbit phospho-Ser19-TH plus mouse proteasome antibodies (lower panel) followed by incubation with an anti-rabbit antibody coupled to Alexa 488 and anti-mouse coupled to AlexaFluor 594. See text for details. Scale bar = 5 μm. Three separate cell culture preparations were performed and 10 different images were taken and analyzed for each experiment. One representative image is presented.

TH activity can be regulated by protein phosphorylation at serine (Ser) residues by diverse protein kinases [30]. Phosphorylation of TH at Ser-19 site has no effect on TH activity, althouth it alters the enzyme conformation allowing increased accessibility of Ser-40 to kinases leading to enhanced enzyme activity [30,31]. As shown in Fig. 4, phosphorylation of TH at Ser-19 and Ser-40 sites was increased by 5.6±1.7 and 2.7±0.3 fold above basal, respectively, when PC12 cells were exposed to 1 μmol/L lactacystin for 30 min, suggesting that the phosphorylated TH is accumulated as a result of proteasome inhibition. In addition, phosphorylated TH at Ser19 or Ser40 colocalized with the proteasome in PC12 cells (Fig. 5).

Altogether these results suggest that TH is short-term regulated by the UPS in PC12 cells.

**Fig 6:**
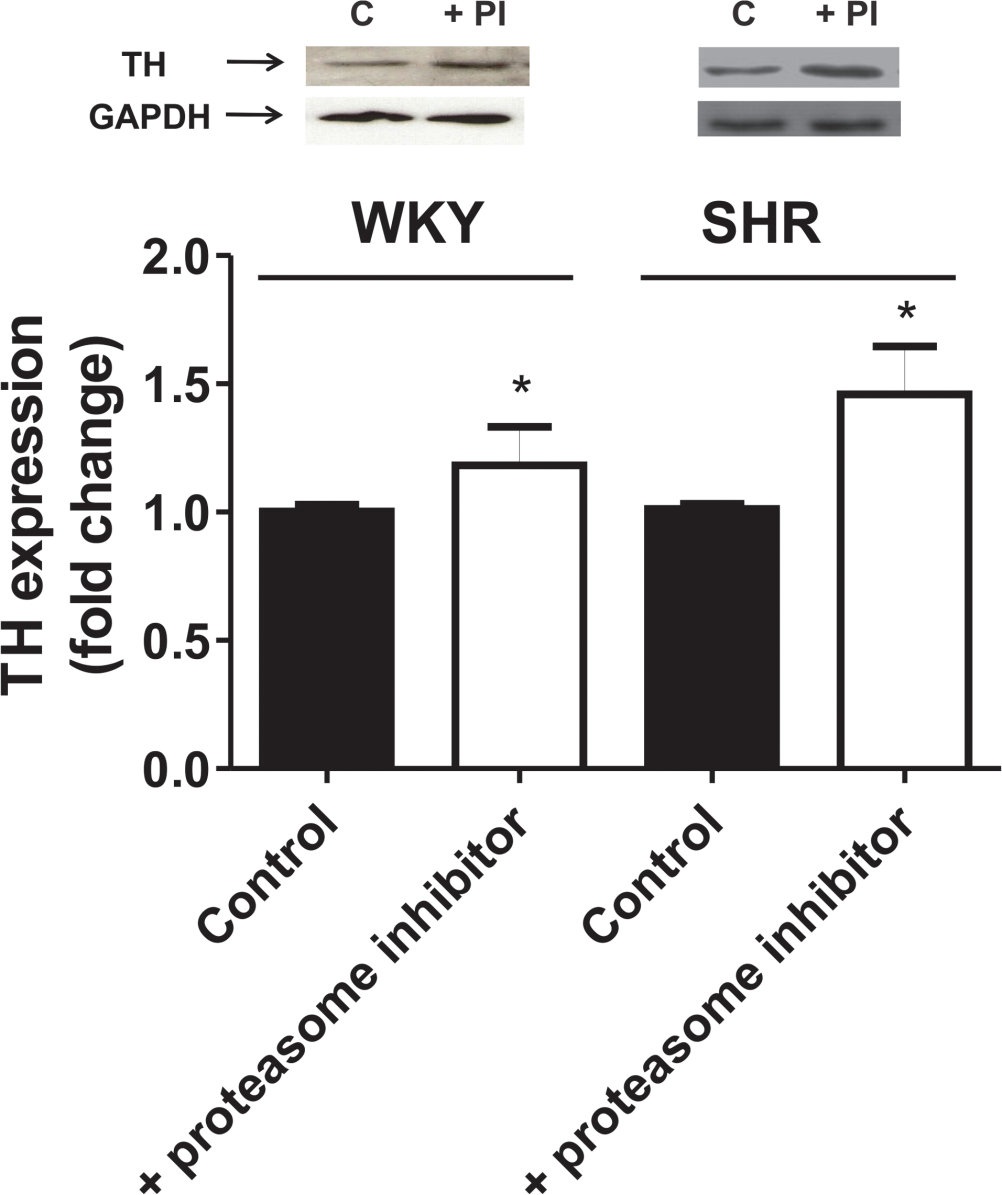
TH protein level in neuronal cultures from hypothalami and brainstem of WKY or SHR incubated in the absence (control) or presence of the proteasome inhibitor Lactacystin (5 μmol/L) (PI) during 30 min. TH protein level was assessed by Western-blot as described under Methods. Blots were stripped and reprobed for GAPDH to assess protein loading. Signals from TH protein level were normalized to GAPDH levels. Each bar represents the mean ± SEM (n = 7). * P<0.05 as compared to each corresponding control.

We also investigated the involvement of the UPS on TH turnover in neurons. Neuronal cultures from the hypothalamus and brainstem of WKY or SHR were exposed to the proteasome inhibitor for 30 min and TH protein content assessed. Results showed that TH protein content was increased above basal in neurons from WKY and SHR when the proteasome was inhibited supporting that TH protein content is effectively centrally regulated by the UPS (Fig. 6).

### Proteasome activity is impaired in hypertension

**Fig 7:**
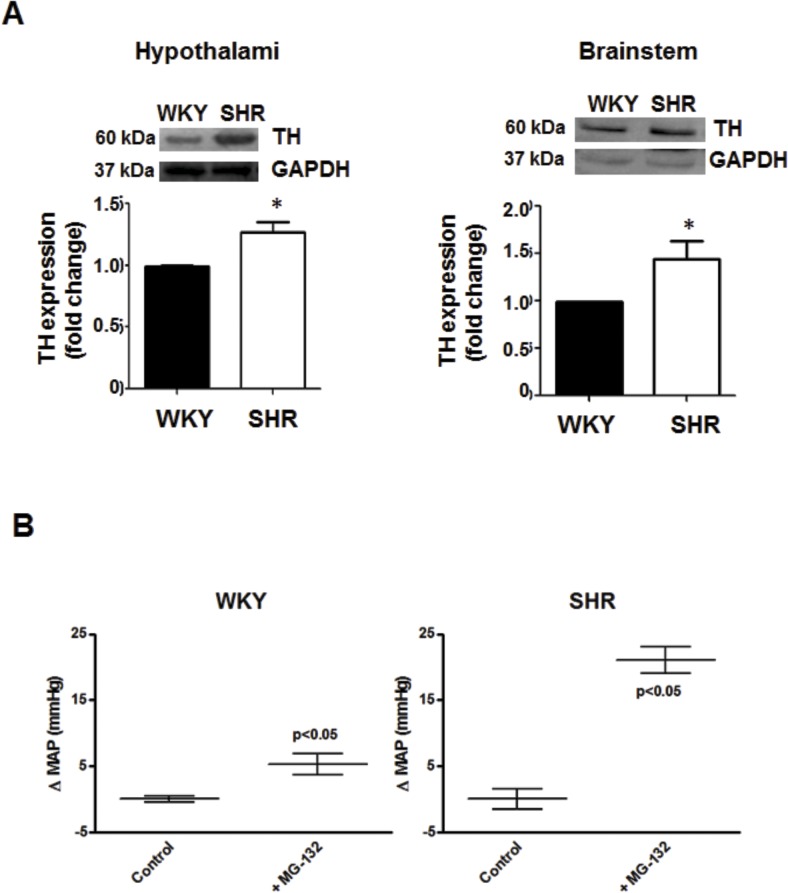
TH protein level in the hypothalamus and brainstem of WKY or SHR rats. A, TH protein level was assessed by Western-blot as described under Methods. Blots were stripped and reprobed for GAPDH to assess protein loading. Signals from TH protein level were normalized to GAPDH levels. Each bar represents the mean ± SEM (n = 7). * P < 0.05 as compared to WKY. B, Change in mean arterial pressure (MAP) in WKY and SHR after intracerebroventricular injection of aCSF (control) or the proteasome inhibitor MG-132. Values are mean ±SEM (n = 7). * P < 0.05 as compared with basal.

As the increase in TH protein content reported by others and us in neurons from SHR [12, 13] may result from impaired UPS activity, we investigated proteasome activity and protein content in SHR brain areas related to blood pressure regulation. Fig. 7A shows that TH protein content was increased in SHR hypothalamus and brainstem compared to its normotensive control. Increased TH may be correlated with enhanced TH activity in the hypothalamus and elevated blood pressure as previously reported in SHR [11, 12]. Since the proteasome regulates TH protein levels, it may consequently affect blood pressure in SHR. Thus, we evaluated the effect of proteasome inhibition on blood pressure. Intracerebroventricular injection of the proteasome inhibitor induced a significant increase in blood pressure in WKY and SHR, being this increase greater in SHR (5.3±1.6 mmHg increase in WKY vs 21.0±2.0 mmHg in SHR) (Fig. 7B). This result shows that proteasome inhibition in WKY rats leads to hypertension reproducing the SHR phenotype.

We next addressed whether the activity and expression of the proteasome were modified in SHR.

**Fig 8:**
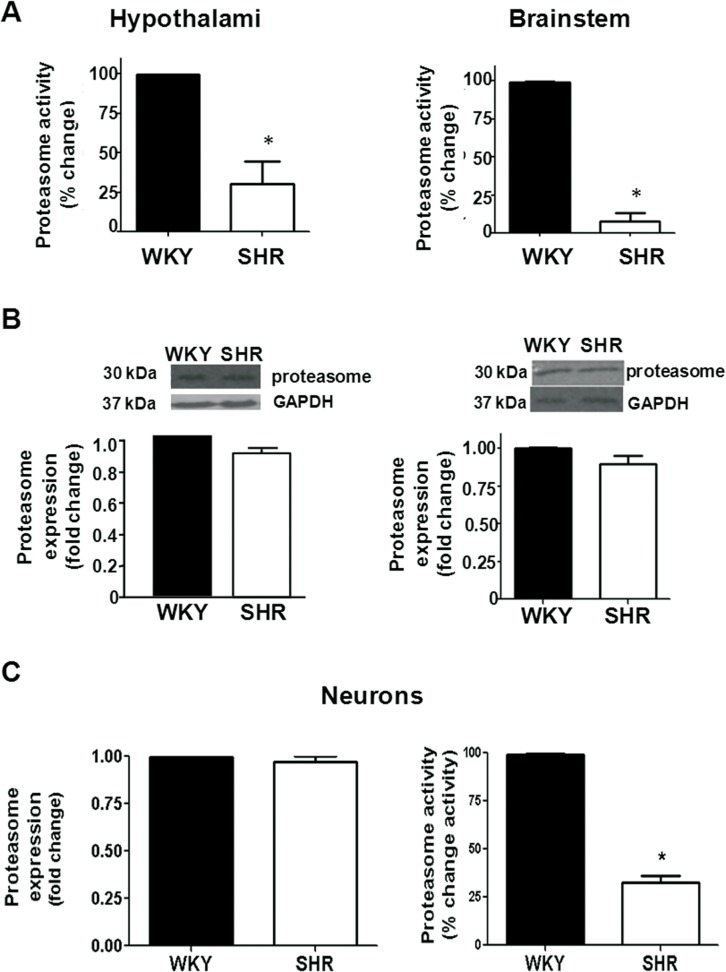
A, Proteasome activity in the hypothalamus and brainstem of WKY and SHR rats. Values are mean ±SEM (n = 7). * P < 0.05 as compared with WKY. B, Proteasome protein level in the hypothalamus and brainstem, respectively, from WKY and SHR. Proteasome protein level was measured by Western-blot as described under Methods. Blots were stripped and reprobed for GAPDH to assess protein loading. Signals from proteasome protein level were normalized to GAPDH levels. Values are mean ±SEM (n = 7). * P < 0.05 as compared to WKY. C, Proteasome protein level and activity in neuronal cultures from the hypothalamus and brainstem of WKY and SHR rats. Values are mean ±SEM of 3 determinations from three separate cell culture preparations. * P < 0.05 as compared with WKY.

Proteasome activity was diminished in the SHR hypothalamus as compared to WKY whereas the protein content was not modified (Fig. 8A). Similarly, proteasome activity was diminished whereas its protein level was not modified in the brainstem of SHR (Fig. 8B). Proteasome activity was also measured in a tissue not involved in blood pressure regulation. Proteasome activity was not significantly different from SHR lungs compared to WKY (data not shown), suggesting that proteasome dysfunction is related to tissues involved in blood pressure control.

Since TH is endogenously expressed in neurons and is increased in hypertension [12,13], we investigated whether the proteasome dysfunction observed in the hypothalamus and brainstem from SHR was also evident in neurons. As it is shown in Fig. 8 (C), proteasome protein content was not modified in neuronal cultures from SHR as compared to its normotensive control, but proteasome activity was significantly reduced by 67±4%.

These findings support a proteasome dysfunction in hypothalamus and brainstem of SHR.

## Discussion

Present findings show that the blockade of the proteasome up-regulates TH protein content as well its phosphorylation at the Ser 40 site, which is essential for full activation of the enzyme. The up-regulation of TH by proteasome inhibition was evident at 30 min and after 12 h whereas by lysosome inhibition it was observed at 6 h suggesting that the proteasome is involved in the short-term regulation of TH whereas lysosomes at longer times. Furthermore, our present work shows that the proteasome activity was decreased in hypothalamic and brainstem neurons of SHR, and this may account for the increase in central TH activity and expression previously reported in SHR [12,13].

TH, the enzyme that catalyzes the first and rate-limiting step in cathecolamines biosynthesis, is localized in presynaptic cathecolaminergic neurons [10]. Several evidence support the relevance of the UPS in neurons [5,32]. The formation and maintenance of specialized neuronal structures and neuronal activity is known to involve local protein degradation controlled by Ub conjugation. A main function of the presynaptic compartment is the release of specific neurotransmitters from presynaptic vesicles into the synaptic cleft upon neuronal activation. Several evidence show that presynaptic processes are also under the control of local ubiquitin-dependent regulation pathways. In this sense, enzymes of the UPS localize to presynaptic boutons in Drosophila, and the inhibition of the 26S proteasome elevates synaptic transmission. One known presynaptic target of the UPS is Dunc13, which is important for synaptic vesicle fusion [32].

TH turnover is around 17 h [15]. In agreement, an increase in TH protein levels was observed when the proteasome was inhibited for more than 12 h (present results). However, present findings also show that TH was short-term regulated by the UPS, given that following 30 min cell exposure to proteasome inhibition, an increase in TH protein level, phosphorylation and ubiquitination, a tag necessary to be recognized for the proteasome, was observed. In accordance, TH short-term regulation by the UPS was also observed in neurons from the hypothalamus and brainstem of SHR (present results). In a previous study, Nakashima et al. [18] reported that no changes in protein levels were observed with proteasome activity blockade. The discrepancy with our study may arise from the different time experimental protocols used. While that study was conducted at two time points (4 and 8 h), our study was performed from 30 min to 24 h. However, at 6 h our results are similar to those reported by Nakashima et al. [18], that is, no TH protein level regulated by the proteasome, because at this time, according to our results, lysosome regulate TH protein content.

Proteins targeted for proteasome degradation are tagged with Ub [3]. A polyubiquitin chain anchored to the substrate has been long stated for proteasomal recognition. However, several recent studies have demonstrated that monoubiquitylation or multiple monoubiquitylation can be sufficient for efficiently targeting certain substrates for proteasomal degradation ([27,28,33]). Proteins not only shorter but also larger than 150 residues can be degraded by the proteasome when monoubiquitylated. For instance, monoubiquitylation of paired box 3, an important regulator of muscle differentiation, or multiple monoubiquitylation events on phospholipase D target these proteins containing more than 150 residues for proteasomal degradation [33]. Our results show that TH is a substrate for the Ub-conjugating enzyme, being mono-ubiquitylated prior to its degradation by the proteasome. In accordance, the mono-ubiquitylation of TH has been shown in the catecholamine storage/secretory granules of the adrenal medulla [34]. Ub-TH has been reported both in vitro as a soluble recombinant human enzyme and in vivo as a membrane-bound form of the enzyme in the catecholamine storage/secretory granules of the adrenal medulla [34]. The integral TH of the bovine adrenal chromafin granule membrane was detected mainly as mono-ubiquitylated. In contrast, multi/poly Ub conjugates were observed for the soluble recombinant human TH in the reticulocyte lysate system [34].

Most degradation by the proteasome depends on ubiquitin conjugation. However, for a significant subset of proteins turnover is independent of ubiquitin conjugation. For instance, Rpn4, a transcriptional regulator of proteasome homeostasis, thymidylate synthase, an enzyme required for production of DNA precursors and ornithine decarboxylase, the initial enzyme committed to polyamine biosynthesis, have been reported to be degraded in a Ub-independent manner [29].

TH activity can be regulated by protein phosphorylation at serine (Ser) residues by a variety of protein kinases [30]. Phosphorylation of TH at Ser-19 has no effect on TH activity but changes the enzyme conformation facilitating the accessibility of Ser-40 to kinases so leading to an increase in the enzyme activity [30,31]. Phosphorylation of Ser-40 results in at least 20-fold activation of TH. Phosphorylation at Ser-31 also increases TH activity but to a much lesser extent than for Ser-40 phosphorylation [30,31,35,36], thus we only measured phosphorylation at Ser-40. Our study shows that proteasome inhibition induced an increase in the levels of TH phosphorylated at Ser19 and Ser40, suggesting that not only TH protein level but also TH activity may be modulated by the UPS. We should point out that in the present study we did not measure TH activity, but instead we evaluated TH phosphorylation as TH activation index measurement. In accordance with our present study, a recent work showed that the inhibition of proteasome activity increases the quantity of TH molecules phosphorylated at their Ser19 and Ser40 [18]. In addition, Kawahata et al. [37] have shown that 12 to 48 h proteasomal inhibition leads to accumulation of insoluble phospho-Ser40-TH and formation of phospho-Ser40-TH-containing intracellular aggregates which tightly co-localized with Ub, suggesting that phospho-Ser40-TH is prone to be insolubilized and aggregated by dysfunction of an UPS in PC12D cells.

The pathological states associated with the UPS can be classified into two groups: 1) those that result from loss of function, mutation in a ubiquitin system enzyme or target substrate, that result in stabilization of certain proteins and 2) those that result from gain of function, abnormal or accelerated degradation of the protein target [1,3]. In the present work a loss of proteasome function was observed in the hypothalamus and brainstem from hypertensive rats and this observation is not only limited to hypertension. Proteasomal dysfunction, which would induce an accumulation and aggregation of cytoplasmic proteins, may be one of the causes of neurodegenerative disorders [1,4,5]. UPS aberrations may contribute not only to the pathogenesis of neurodegenerative disorders, several evidence suggest that insulin-resistance, diabetes, endothelial dysfunction, atherosclerosis and cardiovascular diseases share in common a deregulation of the UPS [6,7,9,38–40]. Taking into account this evidence, the increased TH protein content and activity reported in hypothalamic neurons from SHR [12,13] may be associated with UPS activity impairment. In the present work, a decrease in proteasome activity was observed in neurons from the hypothalamus and brainstem from SHR, despite the fact that proteasome protein level was not changed in this pathological situation. This impairment in proteasome activity may be responsible for the increased TH protein level in SHR. Further, the impairment in the neuronal UPS system observed in this present work may affect the turnover of others proteins, favoring their accumulation. It is important to note that the CNS of SHR displays abnormal features compared to normotensive rats [41]. In the phase of established hypertension these rats develop brain atrophy, loss of nerve cells, and glial reaction, a phenomena shared to some extent with hypertensive brain damage in humans. An altered anatomical organization of hypothalamic regions and gross morphological brain differences between SHR and age-matched normotensive WKY rats were reported. The hippocampus, a key area for learning and memory, is smaller in SHR compared with that in normotensive cohorts, as well as the density of nuclei in the CA1 subfield. In fact, these rats presents memory and learning impairment. From a neurotransmitter point of view, they present changes of some specific neurotransmitter systems that may have functional and behavioral relevance. An impaired cholinergic neurotransmission characterizes SHR, similar to those reported in patients affected by vascular dementia. SHR are also characterized by a dopaminergic hypofunction and noradrenergic hyperactivity similarly as occurs in attention-deficit with hyperactivity disorder [41]. These noradrenergic hyperactivity may be correlated with proteasome dysfunction observed in hypothalamus and brainstem (present results).

In conclusion, present findings show that TH protein level is not only long-term but also short-term regulated by the UPS and that the enzyme is ubiquitylated prior to its degradation by the proteasome. Furthermore, proteasome activity is impaired in SHR. The fact that blood pressure increases in SHR after proteasome inhibition suggest that the decreased proteasome activity is not saturated in SHR, and therefore may not represent the only key mechanism in hypertension. Alterations in the proteasome activity may be one of the causes of the increased TH levels reported in hypertension. Improper regulation of TH content due to malfunctioning of the stabilization mechanism could lead to profound changes in catecholamine levels, thus affecting behavior and cardiovascular responses controlled by the central nervous system.
