# Correction: Tyrosine Hydroxylase Is Short-Term Regulated by the Ubiquitin-Proteasome System in PC12 Cells and Hypothalamic and Brainstem Neurons from Spontaneously Hypertensive Rats: Possible Implications in Hypertension

**DOI:** 10.1371/journal.pone.0130785

**Published:** 2015-06-25

**Authors:** Nadia A. Longo Carbajosa, Gerardo Corradi, María A. Lopez Verrilli, María J. Guil, Marcelo S. Vatta, Mariela M. Gironacci

The first author’s name is cited incorrectly. The correct name is: Nadia A. Longo Carbajosa. The correct citation is: Longo Carbajosa NA, Corradi G, Verrilli MAL, Guil MJ, Vatta MS, Gironacci MM (2015) Tyrosine Hydroxylase Is Short-Term Regulated by the Ubiquitin-Proteasome System in PC12 Cells and Hypothalamic and Brainstem Neurons from Spontaneously Hypertensive Rats: Possible Implications in Hypertension. PLoS ONE 10(2): e0116597. doi:10.1371/journal.pone.0116597
